# Mechanisms of Memory Retrieval in Slow-Wave Sleep

**DOI:** 10.1093/sleep/zsx114

**Published:** 2017-07-06

**Authors:** Scott A Cairney, Justyna M Sobczak, Shane Lindsay, M Gareth Gaskell

**Affiliations:** 1 Department of Psychology, University of York, United Kingdom;; 2 Psychology, School of Life Sciences, University of Hull, United Kingdom

**Keywords:** sleep, memory, reactivation

## Abstract

**Study Objectives:**

Memories are strengthened during sleep. The benefits of sleep for memory can be enhanced by re-exposing the sleeping brain to auditory cues; a technique known as targeted memory reactivation (TMR). Prior studies have not assessed the nature of the retrieval mechanisms underpinning TMR: the matching process between auditory stimuli encountered during sleep and previously encoded memories. We carried out two experiments to address this issue.

**Methods:**

In Experiment 1, participants associated words with verbal and nonverbal auditory stimuli before an overnight interval in which subsets of these stimuli were replayed in slow-wave sleep. We repeated this paradigm in Experiment 2 with the single difference that the gender of the verbal auditory stimuli was switched between learning and sleep.

**Results:**

In Experiment 1, forgetting of cued (vs. noncued) associations was reduced by TMR with verbal and nonverbal cues to similar extents. In Experiment 2, TMR with identical nonverbal cues reduced forgetting of cued (vs. noncued) associations, replicating Experiment 1. However, TMR with nonidentical verbal cues reduced forgetting of both cued and noncued associations.

**Conclusions:**

These experiments suggest that the memory effects of TMR are influenced by the acoustic overlap between stimuli delivered at training and sleep. Our findings hint at the existence of two processing routes for memory retrieval during sleep. Whereas TMR with acoustically identical cues may reactivate individual associations via simple episodic matching, TMR with nonidentical verbal cues may utilize linguistic decoding mechanisms, resulting in widespread reactivation across a broad category of memories.

**Statement of Significance**

Memories can be covertly reactivated in sleep by re-exposing individuals to auditory stimuli encountered at learning; a technique known as targeted memory reactivation (TMR). Studies have shown that TMR enhances memory consolidation, but little is known about the nature of the cognitive mechanisms by which memories are retrieved for reactivation in the sleeping brain. We report on two experiments which demonstrate that the memory effects of TMR are influenced by the degree of acoustic overlap between auditory stimuli presented at learning and in sleep. Our data provide evidence that there are two processing routes for memory retrieval in sleep. These findings are pertinent to our understanding of the mechanisms by which memories are accessed offline in the healthy human brain.

## INTRODUCTION

Memory consolidation, the process by which initially weak and labile memories become strong and enduring representations, is facilitated by sleep.^1–4^ Beyond passively shielding newly learned information from wakeful interference and decay, the sleeping brain is thought to reactivate and strengthen memories for recent experiences.^5,6^ The Active Systems account of sleep and memory consolidation proposes that the cardinal electroencephalographic (EEG) oscillations of slow-wave sleep (SWS), namely slow oscillations (<1 Hz), spindles (~12–15 Hz), and ripples (~80–200 Hz), work in unison to mediate memory reactivations and overnight consolidation.^7^ Memory reactivations therefore promote plasticity, as is necessary for memory reorganization between the hippocampus and neocortical networks.^8–10^

Studies in animals and humans have provided compelling evidence that memories are reactivated in SWS.^11–13^ The recent development of a technique known as targeted memory reactivation (TMR) has made it furthermore possible to covertly retrieve and reactivate individual memories during sleep via olfactory or auditory cues and selectively enhance their consolidation (for review see Oudiette and Paller, Schouten et al.).^14,15^ In a typical auditory TMR experiment, new memories are associated with auditory stimuli at encoding and half of the stimuli are then replayed during SWS. Recall accuracy is typically higher for cued (vs. noncued) memories, indicating that TMR enhances memory processing in sleep. The benefits of auditory TMR for consolidation have been observed across a range of memory domains in humans, including verbal and nonverbal declarative memory,^16–20^ procedural memory,^21–24^ and emotional memory.^25,26^

The clear success of TMR in terms of improving subsequent memory performance implies that auditory stimuli are effective in cueing their associated memories during SWS. In order for this to work, there must be—at least implicitly—a process of memory retrieval: the auditory cue must activate the necessary perceptual mechanisms during SWS so that the relevant recent memory trace can be identified for enhancement. While much of the focus of previous work has been on the memory enhancement aspect of TMR, the memory retrieval aspect is less well understood. The current study is intended to fill this gap.

The majority of auditory TMR studies have employed nonverbal cues such as environmental sounds,^17–20,25^ artificial sounds,^27^ or melodies.^21–24^ Recent work has also shown a memory benefit of TMR with verbal cues in both linguistic^28–30^ and nonlinguistic memory paradigms.^26,31^ Whether the memory effects of TMR with verbal and nonverbal cues are directly comparable, however, is still unknown. This is an important question because it speaks to the way in which memories are retrieved during sleep. Spoken words are the classical examples of arbitrary signs,^32^ meaning that a complex multilevel decoding process is engaged during normal wakeful recognition in order to access meaning.^33^ Environmental sounds, on the other hand, may well have a more direct link to an associated concept. These differing levels of analysis could be important in the sleeping brain in terms of its ability to retrieve newly acquired memories via cueing with verbal and nonverbal stimuli, potentially reducing the scope for memory enhancement via verbal (vs. nonverbal) TMR. Nonetheless, on account of prior work suggesting that some degree of verbal semantic processing is retained during sleep,^34,35^ it is possible that verbal and nonverbal TMR may yield equivalent overnight memory benefits.

A further way in which verbal materials might trigger memory retrieval in sleep would circumvent the usual speech decoding mechanisms. When a spoken word is encountered in the context of an encoding session, a detailed episodic trace of that word will be formed,^36,37^ and this may be sufficient to access the associated memory directly during sleep, bypassing the usual wake-like decoding mechanisms. However, this kind of more direct retrieval would depend on a strong acoustic match between the verbal stimulus heard in the encoding episode and the cue stimulus presented during sleep. In all prior studies of verbal TMR, the spoken word cues delivered in sleep have indeed been identical to those heard at training. Whether verbal TMR with spoken words that are not identical to training (eg, presented in a different voice) can also facilitate consolidation is therefore unknown but important to determine. If wake-like decoding mechanisms are at play during verbal TMR, then the memory effects of nonidentical verbal cues may be akin to those of identical verbal cues.

To summarize, there are two ways in which memories may be retrieved via verbal TMR in sleep. If retrieval depends on wake-like decoding mechanisms, then TMR with verbal cues may yield less effective memory benefits than simpler environmental sound cues. However, such a mechanism would be generalizable, in that the same outcome of verbal TMR should be observed irrespective of whether the cues are presented in the same or a different voice to training. On the other hand, if verbal cues access their associated memories via a more direct acoustic matching process, then spoken words might be just as effective as environmental sounds in TMR but only if the reactivation cue is a strong acoustic match to the encoded stimulus. In other words, this direct route of covert memory retrieval would not generalize well to new speakers.

We addressed these issues in two experiments. In Experiment 1, we compared the effects of TMR with verbal and nonverbal cues on the overnight consolidation of declarative memory. Participants were trained to associate spoken words or sounds with unrelated visual target words before a night of sleep. Subsets of the spoken words (verbal TMR) and sounds (nonverbal TMR) were replayed in SWS before paired-associate recall was assessed in the morning. In Experiment 2, we examined the memory effects of verbal TMR when the spoken word cues were not identical to those encountered at training. To do this, we used the same paradigm as Experiment 1, with the single difference that the gender of the spoken words was switched between training and sleep (the sounds remained identical to training). In both Experiments 1 and 2, each of the target words was presented in a specific screen location, enabling us to also assess the effects of TMR on spatial memory consolidation.

## METHODS

### Stimuli

#### Visually Presented Words

Seventy words were extracted from an adapted version of The University of South Florida (USF) word association, rhyme, and word fragment norms^38,39^ for use as paired-associate targets. The words were divided into two sets (A and B) of 35 items that were matched for concreteness (mean ± standard deviation [SD], A = 5.76 ± 0.62, B = 5.68 ± 0.54, *t*(34) = 0.63; *p = .*54), frequency (mean ± SD, A = 30.37 ± 39.21, B = 29.83 ± 38.31, *t*(34) = 0.06; *p = .*96), and length (mean ± SD, A = 4.94 ± 0.76, B = 4.94 ± 0.84, *t*(34) = 0.00; *p* = 1.00). All words were either monosyllabic or disyllabic (mean number of syllables ± SD, A = 1.34 ± 0.48, B = 1.34 ± 0.48, *t*(34) = 0.00; *p* = 1.00).

#### Auditory Stimuli: Spoken Words

An additional 35 monosyllabic and disyllabic words were extracted from the USF norms for use as spoken words in the paired associates task (mean number of syllables ± SD = 1.54 ± 0.51). In order to test the acoustic specificity of verbal TMR effects, all items were recorded using two separate speakers, one male and one female. The male and female word recordings were matched in duration (mean ± SD ms, male = 769.29 ± 104.95, female = 774.80 ± 99.14, *t*(34) = 0.49; *p = .*63). An additional word (“surface”) was taken from the USF norms for use as a spoken control cue (male version = 990 ms; female version = 950 ms). The abstract nature of this control word was intentional so that it remained distinct from the study words.

#### Auditory Stimuli: Environmental Sounds

Thirty-five environmental sounds were adopted from two prior studies of memory reactivation in sleep^17,18^ and freesound.org. The sounds were similar in length to both the male and female versions of the spoken word cues (mean ± SD = 740.97 ± 156.29, *F*(2,102) = 0.76; *p = .*47). An additional control sound (guitar strum, 524 ms) was adopted from the study by Rudoy et al.^18^

#### Paired Associates

Each visual target word in sets A and B was paired with a spoken word and sound, resulting in two 35-item sets of “speech-word pairs” and two additional 35-item sets of “sound-word pairs”. None of these pairs contained a clear semantic link. During the experiments, the speech-word pairs were taken from one set (eg, set A) while the sound-word pairs were taken from the other set (eg, set B), and this was counterbalanced across participants.

### Experiment 1

#### Participants

Thirty-seven healthy male participants aged 18–24 years were recruited for Experiment 1 and were each paid £30. Nine of these participants were excluded for the following reasons: inability to reach SWS in the first half of the night (2), repeated arousals or awakening during TMR (4), inability to reach the recall performance criteria within an allotted four test rounds (2), and computer malfunction (1). This left analysis of data from the remaining 28 participants aged 18–24 years (mean ± SD age, 20.32 ± 1.54 years). Prestudy screening questionnaires indicated that participants had no history of sleep, psychiatric, or neurological disorders; were not using any psychologically active medications; had not consumed alcohol or caffeine during the 24 hours that preceded the study; and were nonsmokers. As evaluated with the Pittsburgh Sleep Quality Index,^40^ all participants had obtained a normal pattern of sleep across the month preceding the study. Written informed consent was obtained from all participants in line with the Research Ethics Committee of the Department of Psychology, University of York, who approved the study.

#### Procedure

**Figure 1:**
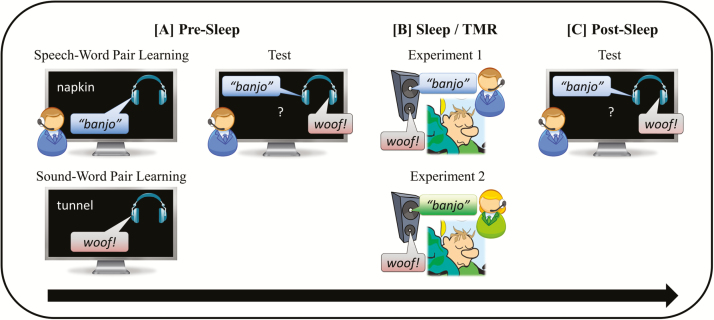
Experimental procedures. (A) Presleep (09.30 pm): Speech-word pairs and sound-word pairs (and associated screen locations) were encoded separately. Participants then carried out a presleep test for all speech-word and sound-word pairs. (B) Sleep/TMR (11.00 pm–07.00 am): sounds and spoken words were replayed throughout the first two cycles of slow-wave sleep. In Experiment 1, the spoken words were presented in a male or female voice at training and replayed in the same voice in sleep. In Experiment 2, the spoken words were presented in a male voice at training and replayed in a female voice in sleep. The replayed sounds were identical to the stimuli presented at training in both experiments. (C) Post sleep (07.30 am): participants completed a post-sleep test, which was identical to the presleep test. TMR = targeted memory reactivation.

An overview of the core experimental procedures and tasks is presented in Figure 1. The experiment began at 09.30 pm (± 30 minutes) and was carried out in the Sleep, Language and Memory Laboratory, Department of Psychology, University of York. Two experimental sessions were separated by a period of overnight sleep. Participants were informed that they were taking part in a study of memory and sleep but were unaware that TMR would be used during the sleep phase. Before the first session, electrodes were attached to each participant’s scalp and face such that sleep could be monitored with polysomnography (PSG). A detachable electrode board was removed from the main PSG system and fastened across the participant’s chest, enabling them to move around the laboratory with the electrodes in place. Immediately before the first session, participants recorded their self-reported alertness levels using the Stanford Sleepiness Scale.^41^

#### Session 1: Presleep

The first part of this session was divided into two separate sections: training for the speech-word pairs and training for the sound-word pairs, both of which included a learning phase and a test phase. The order of these sections was counterbalanced across participants. In the learning phase, each trial began with a black fixation cross placed in the center of a PC screen for 1500 ms. The fixation cross then turned blue to indicate the onset of an auditory stimulus and, following a delay of 500 ms, a randomly selected spoken word (speech-word pair training) or sound (sound-word pair training) was presented. Spoken words were presented in a male or female voice (counterbalanced across participants). After 1500 ms, a semantically unrelated word appeared in one of the four quadrants of the screen (top/bottom, left/right) for 5000 ms. To facilitate learning, participants were instructed to form a mental image of the visually presented word and auditory stimulus interacting. The learning phase of both speech-word pair training and sound-word pair training consisted of 35 trials: 3 practice trials, 28 experimental trials, and 4 filler trials divided between the beginning and end of the task to serve as respective primacy and recency buffers.^2^ The 28 experimental trials of each learning phase were equally distributed across the four quadrants of the screen, with seven trials appearing in each quadrant. Participants were informed that a memory test would follow immediately after learning. They were also told that their performance assessment would relate to memory for the words and not the locations but were asked to still pay attention to the quadrant of the screen that each word appeared.

In the test phase, each trial began with a black fixation cross placed in the center of the screen for 1500 ms, which then turned blue for 500 ms before a randomly selected spoken word (speech-word pair training) or sound (sound-word pair training) was presented. After 500 ms, the fixation cross was replaced by a rectangular box and participants were instructed to type the target word associated with the auditory stimulus within a time limit of 12 seconds. Responses were finalized via an Enter key press. Participants were informed that all word responses had to be singular, in lower case, and spelled correctly. Corrections could be made with the Backspace key before a response was provided. Immediately after making their response, participants were asked to indicate which quadrant of the screen the word had appeared by pressing the corresponding key on the keyboard number pad (1 = bottom left, 3 = bottom right, 7 = top left, 9 = top right) within 5 seconds. The test phase of both speech-word pair training and sound-word pair training consisted of 31 trials: 3 practice trials, which corresponded to those seen at the learning phase and 28 experimental trials. If participants did not correctly recall >60% of the words associated with the auditory stimuli, they repeated the learning and test phases until this criterion was met. If this criterion was not met within four rounds of testing, participants were excluded from the study.

After completing both speech-word training and sound-word training, participants completed a final presleep test, which provided a baseline index of memory recall for the speech-word pairs and sound-word pairs. This final test followed the same procedures as the test phase described above, except that all 56 experimental items (28 speech-word pairs and 28 sound-word pairs) were included in random order. The six practice trials (three speech-word pairs and three sound-word pairs) were also included at the beginning of this test such that the total number of trials was 62. We informed participants that they would complete this test again in the morning after sleep with the expectation that this knowledge would increase the salience attributed to the learned material and thereby enhance sleep-dependent consolidation.^16,42^

#### TMR Setup

Because our aim was to examine the effects of TMR on forgetting over sleep, we first excluded all of the sound-word pairs and speech-word pairs that were scored as incorrect in the presleep test. Of the correct items, half of the associated spoken words and half of the associated sounds were randomly selected for respective verbal and nonverbal TMR and then intermixed in a randomized order for replay in SWS. The remaining speech-word pairs and sound-word pairs served as controls (ie, no TMR) in the respective verbal and nonverbal conditions. For example, if 22 speech-word pairs were correctly recalled and 18 sound-word pairs were correctly recalled, there would be 11 spoken words in the verbal TMR set and 9 sounds in the nonverbal TMR set (with those remaining serving as 11 verbal no-TMR controls and 9 nonverbal no-TMR controls, respectively). This approach ensured that presleep performance was identical for items that were cued and not cued in sleep and controlled for interindividual differences in presleep learning.^16^ The maximum number of auditory stimuli that could be allocated for TMR (ie, if participants correctly recalled all 56 paired associates) was 28 (14 spoken words and 14 sounds). Employing a performance criterion of >60% correct during the presleep tests thus ensured that a sufficient number of auditory stimuli would be available for the TMR versus no TMR comparison. On occasions where an odd number of speech-word pairs and/or sound-word pairs were correctly recalled in the presleep test, the additional item was either included in the TMR set or used as a control, and this assignment was counterbalanced across the relevant participants. In all cases, the sounds and spoken words used for TMR in Experiment 1 were identical to those presented at training.

#### Sleep and TMR

At ~11.00 pm, participants went to bed and were left to sleep. To habituate participants to auditory stimulation during sleep, background white noise was played via a speaker above the bed at an unobtrusive sound pressure level of 39 dB throughout the sleep period.^19^ TMR began after participants had exhibited at least 2 minutes of sustained SWS (as determined via online PSG monitoring). To promote acoustic clarity, white noise intensity was lowered during the replay of each item. Interspersed within the TMR stimuli were two auditory controls, namely the spoken word “surface” and the sound of a guitar strum. These were played the same number of times that their corresponding verbal and nonverbal cues were replayed, and once each at the beginning of the TMR set to ensure that participants’ sleep would not be disturbed during auditory stimulation. The stimulation rate was one cue every 5 seconds. However, because each participant’s TMR set differed with regard to the number of verbal and nonverbal items, null events were also randomly interspersed between the cues to maintain a total stimulation time of 290 seconds per TMR set. The TMR set was repeatedly replayed throughout the first two cycles of SWS with a 1-minute interval separating each repetition.^17^ The cues were immediately stopped if participants left SWS or showed signs of microarousal or awakening but restarted if they returned to SWS. Participants were woken up at approximately 07.00 am, unless they were exhibiting SWS or rapid eye movement sleep (REM), in which case they were allowed to sleep until either awakening or entering sleep stage I or II. To attenuate the effects of sleep inertia, participants were given a break of ~20 minutes after waking, during which the PSG electrodes were removed.

#### Session 2: Post Sleep

Participants completed the Stanford Sleepiness Scale^41^ for a second time before carrying out a post-sleep test that was identical to the final presleep test (ie, for all speech-word and sound-word pairs). Afterward, participants were informed of the true purpose of the experiment and asked if they had been aware of any auditory stimuli during the sleep period. Participants then completed an auditory cue discrimination task in which they were represented with all 56 experimental auditory stimuli (28 spoken words, 28 sounds) and, for each, asked to indicate via keyboard press whether they thought it had or had not been replayed in sleep.

### Experiment 2

#### Participants

Thirty healthy male participants aged 18–27 years were recruited for Experiment 2 and were each paid £30. Seven of these participants were excluded for the following reasons: inability to reach SWS in the first half of the night (1), repeated arousals or awakening during TMR (4), inability to reach the recall performance criteria within an allotted four test rounds (2). This left analysis of data from the remaining 23 participants aged 18–27 years (mean ± SD age, 20.96 ± 2.38 years). Participants met the same criteria as those of Experiment 1.

#### Procedure

The experimental procedures were the same as those outlined in Experiment 1, with the single difference that the spoken words were always heard in a male voice at training and test but replayed in a female voice during sleep and in the discrimination task. The sound cues replayed in sleep/the discrimination task remained identical to those presented at training and test. Only female spoken word cues were used for TMR in Experiment 2 as Experiment 1 revealed a nonsignificant memory advantage (forgetting score: noncued items – cued items; see data analysis section below) following TMR with female (vs. male) spoken words (*t*(26) = 1.22, *p* = .23).

### Equipment

#### Experimental Tasks

All of the experimental tasks were implemented on a PC with E-Prime version 2.0 (Psychology Software Tools, Inc.). Auditory stimuli were presented via headphones (Beyerdynamic DT 234 PRO) while visual stimuli were presented ~0.5 m from participants on a 23” flat screen LCD monitor (resolution = 1920 × 1080 pixels) positioned at eye level.

#### Polysomnography

An Embla N7000 PSG system with RemLogic version 3.4 software was used to monitor sleep. After the scalp was cleaned with NuPrep exfoliating agent (Weave and Company), gold-plated electrodes were attached using EC2 electrode cream (Grass Technologies). EEG scalp electrodes were attached according to the international 10–20 system at six standardized locations: frontal (F3, F4); central (C3, C4); and occipital (O1, O2), and each was referenced to an electrode on the contralateral mastoid (A1 or A2). Left and right electrooculography electrodes were attached, as were electromyography electrodes at the mentalis and submentalis bilaterally, and a ground electrode was attached to the forehead. Each electrode had a connection impedance of <5 kΩ, and all signals were digitally sampled at 200 Hz. Online sleep scoring was conducted on the referenced central electrodes (C3–A2 and C4–A1). Subsequent offline scoring was carried out in accordance with the criteria of the American Academy of Sleep Medicine^43^ and confirmed that TMR had taken place in SWS. Sleep data scored offline was partitioned according to the percentage of total sleep time spent in sleep stage I, stage II, SWS, and REM.

PSG epochs scored as either stage II or SWS were extracted from all six EEG channels for spindle analysis. Artifacts were then rejected from the data using EEGLAB version 10.0 before a linear finite impulse response filter was used to bandpass filter each channel at 12–15 Hz. An automated detection algorithm^44^ counted discrete spindle events as amplitude fluctuations within the filtered time series that exceeded a threshold of eight times the mean channel amplitude. Spindle density (counts per minute) was then calculated on all referenced EEG channels (F3–A2, F4–A1, C3–A2, C4–A1 O1–A2, O2–A1). Several studies have used this method to probe the role of spindles in sleep-dependent memory consolidation.^19,25,45–48^

#### Targeted Memory Reactivation

TMR was implemented with Presentation version 17.0 (Neurobehavioral Systems, Inc.). Auditory cues were played via a speaker mounted ~1.5m above the bed, which was connected to an amplifier in a separate control room.

### Data Analyses

#### Behavior

To control for differences in presleep recall, we calculated for each participant the proportion of speech-word pairs and sound-word pairs that were correctly recalled in the presleep test but forgotten in the post-sleep test. In both experiments, these paired associates forgetting scores were applied to a 2 (TMR: On/Off) × 2 (Type: Verbal/Nonverbal) repeated measures analysis of variance (ANOVA).

We subtracted the forgetting score for items cued by TMR from the forgetting score for items that were not cued by TMR to create TMR indices for speech-word pairs and sound-word pairs. A positive TMR index indicated that forgetting rates were lower for cued items relative to noncued items, whereas a negative TMR index indicated the opposite.

Spatial memory analyses only included locations for which the associated speech-word pair or sound-word pair had been correctly recalled before sleep. We calculated the proportion of locations that were correctly recalled in the presleep test but forgotten in the post-sleep test. Location forgetting scores were applied to the same analyses as described above for paired associates.

#### Polysomnography

Relevant TMR indices were correlated with time spent in each stage of sleep (Stage I, Stage II, SWS, REM) and sleep spindle density averaged across all EEG channels (F3, F4, C3, C4, O1, O2). Correlations between sleep stages and behavioral measures underwent traditional Bonferroni correction for multiple comparisons α (0.05)/number of tests (8) < 0.006.^45^ Correlations with spindle density were corrected in the same manner (α [0.05]/number of tests [2] < 0.025).

## RESULTS

### Experiment 1

#### Alertness

Self-reported ratings of alertness^41^ were comparable before and after sleep (mean ± standard error of the mean [SEM], presleep = 3.21 ± 0.19; post-sleep = 2.86 ± 0.12, *t*(27) = 1.51, *p = .*14). There was also no significant correlation between SWS time and mean response times for paired associates recall (*r =* 0.06; *p = .*75) or location recall (*r =* 0.02; *p = .*91) after sleep, suggesting that behavioral effects were not influenced by differences in homeostatic sleep pressure.^49–51^

#### TMR Cues

The number of correctly recalled items that were assigned to the TMR set before sleep ranged from 17 (minimum spoken words = 7; minimum sounds = 8) to 26 (maximum spoken words = 14; maximum sounds = 14). Mean ± SD number of cues = 22.82 ± 2.71 (spoken words = 11.57 ± 1.97; sounds = 11.25 ± 1.76).

#### Paired Associates

Before sleep, participants required fewer test rounds to reach the recall performance criterion ( > 60%) for speech-word training (mean ± SEM, 1.32 ± 0.10) than sound-word training (mean ± SEM, 1.79 ± 0.11, *t*(27) = 3.86; *p = .*001). Importantly, an initial 2 (Test: Presleep/Post Sleep) × 2 (Pair: Speech-Word/Sound-Word) repeated measures ANOVA conducted on paired associates recall scores revealed no main effect of Pair (*F*(1, 27) = 0.40; *p = .*53), no main effect of Test (*F*(1, 27) = 0.08; *p = .*79), and no interaction between factors (*F*(1, 27) = 0.10; *p = .*76). This indicates that recall performance was equivalent for speech-word pairs and sound-word pairs across the presleep and post-sleep tests. See Table 1A for paired associates recall data.

**Table 1:** Paired Associates.

	[A] Recall (%)			[B] Forgetting (%)		
	Pair type	Presleep	Post sleep	TMR type	Cued	Not cued
Experiment 1	Sound word	79.85 ( ± 2.55)	79.85 ( ± 2.44)	Nonverbal	2.24 (± 0.92)	5.00 (± 1.23)
	Speech word	81.89 ( ± 2.69)	82.27 ( ± 2.45)	Verbal (same)	1.23 (± 0.59)	3.93 (± 1.20)
Experiment 2	Sound word	81.83 ( ± 1.98)	81.99 ( ± 1.91)	Nonverbal	2.53 (± 0.95)	5.42 (±1.22)
	Speech word	84.32 ( ± 2.75)	85.56 ( ± 2.63)	Verbal (switch)	0.70 (± 0.48)	0.33 (± 0.33)

Data are shown as mean (± SEM). [A] Presleep and post-sleep recall for the sound-word pairs and speech-word pairs. [B] The proportion of items that were correctly recalled in the presleep test but forgotten in the post-sleep test. The gender of the spoken word cues was the same as the training stimuli in Experiment 1 but switched between training and sleep in Experiment 2.

**Figure 2:**
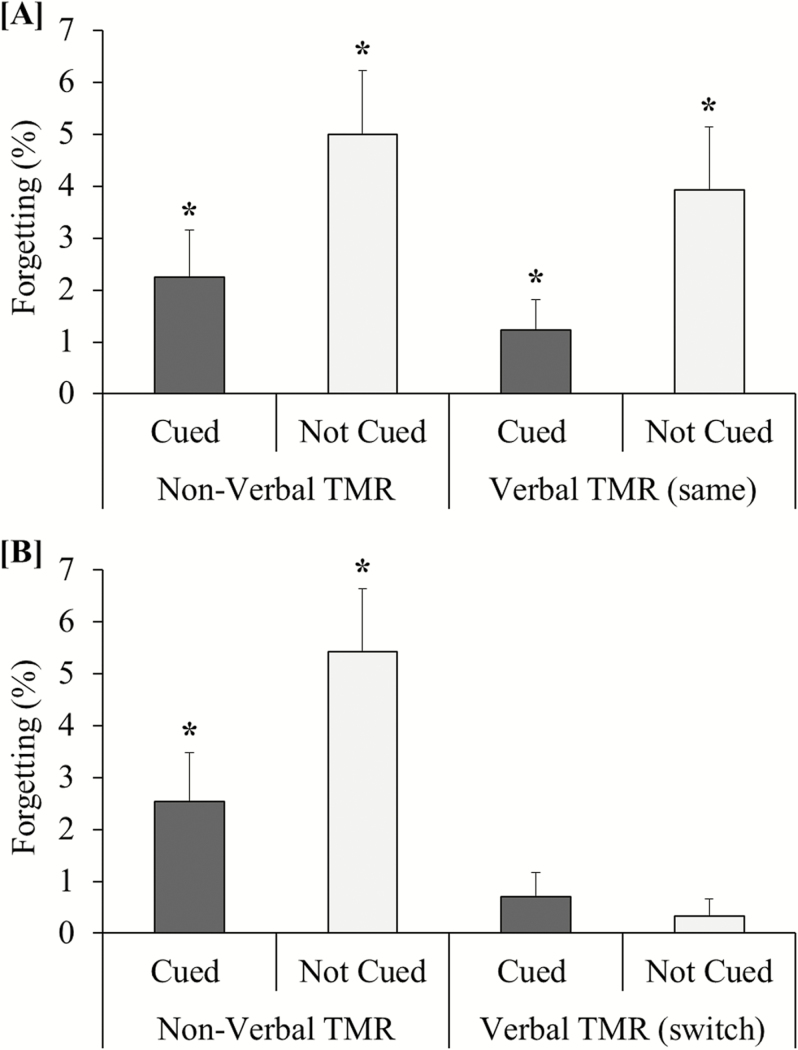
Paired associates forgetting. (A) Experiment 1 and (B) Experiment 2: the proportion of paired associates that were correctly recalled in the presleep test but forgotten in the post-sleep test. The gender of the spoken word cues was the same as the training stimuli in Experiment 1 but switched between training and sleep in Experiment 2. Error bars represent SEM. *Forgetting rates significantly greater than 0 (*p* < .05). TMR = targeted memory reactivation..

Paired associate forgetting scores (ie, the proportion of items that were correctly recalled in the presleep test but forgotten in the post-sleep test) were applied to a 2 (TMR: On/Off) × 2 (Type: Verbal/Nonverbal) repeated measures ANOVA. A main effect of TMR (*F*(1, 27) = 6.85; *p= .*014, *Ƞ*_*p*_^*2*^ = 0.20) indicated that cued paired associates were forgotten to a lesser extent than noncued paired associates. However, there was no interaction between factors (*F*(1, 27) = 0.001; *p = .*97), suggesting that forgetting was reduced by verbal and nonverbal TMR to equal extents (see Figure 2A). There was no main effect of Type (*F*(1, 27) = 0.95; *p = .*34). The mean (± SD) number of full TMR set replays was 6.64 (± 3.03), but this showed no significant relationship with the TMR index (forgetting score: noncued items – cued items) for speech-word pairs (*r* = 0.20; *p = .*31) or sound-word pairs (*r* = −0.12; *p = .*55). See Table 1B for paired associates forgetting data.

#### Locations

Our analyses only included locations for which the associated speech-word pair or sound-word pair was correctly recalled in the presleep test. A 2 (Test: Presleep/Post Sleep) × 2 (Pair: Speech-Word/Sound-Word) repeated measures ANOVA conducted on location recall scores revealed no main effect of pair (*F*(1, 27) = 1.43; *p = .*24), no main effect of test (*F*(1, 27) = 0.63; *p = .*43), and no interaction between factors (*F*(1, 27) = 0.00; *p* = 1.00). This indicates that location recall was equivalent for associated speech-word pairs and sound-word pairs across the immediate and delayed tests. See Table 2A for location recall data.

**Table 2:** Locations.

	[A] Recall (%)			[B] Forgetting (%)		
	Associated pair type	Presleep	Post sleep	TMR type	Cued	Not cued
Experiment 1	Sound word	60.08 (± 4.65)	59.57 (± 4.58)	Nonverbal	16.32 (± 5.13)	19.42 (± 5.67)
	Speech word	55.61 (± 4.49)	55.10 (± 4.50)	Verbal (same)	19.72 (± 4.86)	18.47 (± 5.76)
Experiment 2	Sound word	50.62 (± 4.57)	51.09 (± 3.83)	Nonverbal	21.25 (± 4.11)	20.49 (± 3.74)
	Speech word	46.27 (± 4.57)	42.86 (± 4.98)	Verbal (switch)	21.76 (± 3.89)	21.83 (± 4.33)

Data are shown as mean (± SEM). Analyses only included locations for which the associated speech-word pair or sound-word pair was correctly recalled in the presleep test. [A] Presleep and post-sleep recall for locations associated with sound-word pairs and speech-word pairs. [B] The proportion of locations that were correctly recalled in the presleep test but forgotten in the post-sleep test. The gender of the spoken word cues was the same as the training stimuli in Experiment 1 but switched between training and sleep in Experiment 2.

Location forgetting scores were applied to a 2 (TMR: On/Off) × 2 (Type: Verbal/Nonverbal) repeated-measures ANOVA. There was no main effect of TMR (*F*(1, 27) = 0.10; *p= .*76), indicating that cued and noncued locations were forgotten at similar rates. There was also no main effect of type (*F*(1, 27) = 0.25; *p = .*62) and no interaction between factors (*F*(1, 27) = 0.80; *p = .*38). See Table 2B for location forgetting data.

#### Sleep Stages and Spindle Density

Sleep stage and spindle density data can be found in Tables 3 and 4, respectively. The TMR index for sound-word pairs (ie, nonverbal TMR) was correlated with time spent in REM (*r =* 0.41; *p = .*03), but this relationship did not survive correction for multiple comparisons. TMR indices were not significantly correlated with time spent in any other stage of sleep or spindle density averaged across all EEG channels (all *p > .*05).

**Table 3:** Sleep Stages.

	TST (min)	Stage I	Stage II	SWS	REM
Experiment 1	437.55 ( ± 6.03)	11.76 ( ± 0.98)	52.41 ( ± 1.14)	18.27 ( ± 1.00)	17.55 ( ± 0.81)
Experiment 2	437.89 ( ± 9.99)	12.11 ( ± 1.04)	52.03 ( ± 1.35)	15.80 ( ± 1.03)	20.04 ( ± 0.97)

Data are shown as mean (± SEM). Percentage of total sleep time (TST) spent in each stage of sleep.

REM = rapid eye movement sleep; SWS = slow-wave sleep.

**Table 4:** Spindle Density.

	Mean	F3	F4	C3	C4	O1	O2
Experiment 1	0.66 ( ± 0.05)	1.03 ( ± 0.09)	0.92 ( ± 0.08)	0.81 ( ± 0.09)	0.66 ( ± 0.07)	0.27 ( ± 0.04)	0.29 ( ± 0.03)
Experiment 2	0.61 ( ± 0.06)	1.08 ( ± 0.10)	0.95 ( ± 0.09)	0.65 ( ± 0.07)	0.56 ( ± 0.06)	0.22 ( ± 0.03)	0.23 ( ± 0.03)

Data are shown as mean ± SEM. Sleep spindle density (counts per minute, 12–15 Hz) for each EEG channel.

#### Discrimination Task

None of the participants claimed to have been aware of the auditory stimuli during the night. Because all stimuli assigned to the TMR and no TMR conditions were correctly recalled before sleep, the number of cued stimuli was always substantially smaller than the number of noncued stimuli (as the noncued items included those that were forgotten in the presleep test). To compensate for this, our analysis of the discrimination task data was also restricted to paired associates that participants had correctly recalled in the presleep test. The number of auditory stimuli that participants had guessed were replayed in sleep (mean ± SEM, cued sounds = 5.96 ± 0.42; noncued sounds = 5.57 ± 0.39; cued spoken words = 5.57 ± 0.42; noncued spoken words = 5.32 ± 0.49) were applied to a 2 (TMR: On/Off) × 2 (Type: Verbal/Nonverbal) repeated-measures ANOVA. There was no main effect of TMR (*F*(1, 27) = 1.28; *p =* .27), no main effect of Type (*F*(1, 27) = 0.39; *p =* .54), and no interaction between factors (*F*(1, 27) = 0.05; *p =* .82), indicating that participants were unable to categorize cued and noncued stimuli correctly.

### Experiment 2

#### Alertness

Alertness scores^41^ were comparable before and after sleep (mean ± SEM, presleep = 2.83 ± 0.21; post sleep = 2.57 ± 0.22, *t*(22) = 0.83, *p = .*42). Again, there was no significant correlation between SWS time and mean response times for paired associates recall (*r* = −0.36; *p = .*09) or location recall (*r* = −0.19; *p = .*37) after sleep.

#### TMR Cues

The number of correctly recalled items that were assigned to the TMR set before sleep ranged from 18 (minimum spoken words = 8; minimum sounds = 8) to 28 (maximum spoken words = 14; maximum sounds = 14). Mean ± SD number of cues = 23.17 ± 2.29 (spoken words = 11.65 ± 1.82; sounds = 11.52 ± 1.44).

#### Paired Associates

Participants again required fewer test rounds to reach the presleep performance criterion for speech-word training (mean ± SEM, 1.39 ± 0.12) than sound-word training (mean ± SEM, 1.74 ± 0.09, *t*(22) = 2.34; *p = .*029). As before, however, a 2 (Test: Presleep/Post Sleep) × 2 (Pair: Speech-Word/Sound-Word) repeated-measures ANOVA conducted on paired associates recall scores revealed no main effect of Pair *F*(1, 22) = 0.79; *p = .*38), no main effect of Test (*F*(1, 22) = 1.56; *p = .*23), and no interaction between factors (*F*(1, 22) = 1.00; *p = .*33). We repeated this ANOVA with an additional between subjects factor “Experiment” (1/2), for which there was no main effect (*F*(1, 49) = 1.05; *p* = .31) and no interaction with any other factor(s) (all *p* > .05), implying that memory performance was consistent between the experimental groups. Presleep paired associates scores were further assessed in a 2 (Experiment: 1/2) × 2 (Pair: Speech-Word/Sound-Word) mixed ANOVA. There was no main effect of Experiment (*F*(1, 49) = 0.73; *p* = .40), no main effect of Pair (*F*(1, 49) = 0.81; *p* = .37), and no interaction between factors (*F*(1, 49) = 0.008; *p* = .93), demonstrating that presleep performance was equivalent across the speech- and sound-word pairs and both experiments.

Paired associates forgetting scores were applied to a 2 (TMR: On/Off) × 2 (Type: Verbal/Nonverbal) repeated-measures ANOVA. The main effect of TMR did not reach statistical significance on this occasion (*F*(1, 22) = 2.88; *p = .*10). However, there was a significant interaction between factors (*F*(1, 22) = 7.11; *p = .*014, *Ƞ*_*p*_^*2*^ = 0.24), implying that forgetting was affected by verbal and nonverbal TMR in different ways (see Figure 2B). Post hoc comparisons revealed that forgetting rates were lower for cued (vs. noncued) sound-word pairs (*t*(22) = 2.37; *p = .*027) but equivalent for cued and noncued speech-word pairs (*t*(22) = 0.60; *p = .*56). There was also a main effect of Type (*F*(1, 22) = 13.20; *p = .*001): overall forgetting rates were lower for speech-word pairs than sound-word pairs. Moreover, forgetting rates for the speech-word pairs were not significantly greater than zero in either the cued (*t*(22) = 1.45; *p* = .16) or noncued condition (*t*(22) = 1.00; *p* = .33). Forgetting rates in all other conditions of experiments 1 and 2, by contrast, were significantly above zero (*p* < .05). Together, these results suggest that verbal TMR with nonidentical cues to training may have diminished forgetting of both cued and noncued speech-word pairs. The mean (± SD) number of full TMR set replays was 5.52 (± 2.52), but this was not significantly correlated with the TMR index for speech-word pairs (*r* = −0.08; *p = .*73) or sound-word pairs (*r =* 0.10; *p = .*65).

To demonstrate that the findings of experiments 1 and 2 were differentiated by the effects of verbal TMR on noncued speech-word pairs, the speech-word forgetting scores were applied to a 2 (Experiment: 1/2) × 2 (TMR: On/Off) mixed ANOVA. This revealed a significant interaction (*F*(1, 49) = 4.45; *p = .*04), with Experiment 1 (vs. Experiment 2) showing greater forgetting of noncued speech-word pairs (*t*(49) = 2.65; *p* = .011) but not cued speech-word pairs (*t*(49) = 0.69; *p* = .50). The extent of these differences also resulted in a main effect of Experiment (*F*(1, 49) = 6.08; *p = .*017), such that overall forgetting rates for speech-word pairs were higher in Experiment 1 than Experiment 2. There was no main effect of TMR (*F*(1, 49) = 2.59; *p = .*11). The same ANOVA conducted with sound-word forgetting scores did reveal a main effect of TMR (*F*(1, 49) = 8.52; *p = .*005), as expected. However, there was no main effect of Experiment (*F*(1, 49) = 0.09; *p = .*77) and no interaction between factors (*F*(1, 49) = 0.005; *p = .*95).

#### Locations

A 2 (Test: Presleep/Post Sleep) × 2 (Pair: Speech-Word/Sound-Word) repeated-measures ANOVA conducted on location recall scores revealed no main effect of Pair (*F*(1, 22) = 2.50; *p = .*13), no main effect of Test *F*(1, 22) = 1.23; *p = .*28), and no interaction between factors *F*(1, 22) = 2.09; *p = .*16). We repeated this ANOVA with the additional between subjects factor “Experiment” (1/2), for which there was no significant main effect (*F*(1, 49) = 2.92; *p* = .09) and no interaction with any other factor(s) (all *p* > .05).

Location forgetting scores were applied to a 2 (TMR: On/Off) × 2 (Type: Verbal/Nonverbal) repeated measures ANOVA. There was no main effect of TMR (*F*(1, 22) = 0.48; *p = .*50), no main effect of Type (*F*(1, 22) = 0.04; *p = .*84), and no interaction between factors (*F*(1, 22) = 0.87; *p = .*36).

#### Sleep Stages and Spindle Density

The TMR index for sound-word pairs (ie, nonverbal TMR) was inversely correlated with time spent in SWS (*r* = −0.53; *p = .*009), but this relationship did not survive multiple comparisons correction. TMR indices were not significantly correlated with time spent in any other stage of sleep or spindle density averaged across all EEG channels (all *p > .*05). There were no significant differences between Experiments 1 and 2 in terms of total sleep time, time spent in any particular stage of sleep or spindle density (all *p* > .05).

#### Discrimination Task

None of the participants claimed to have been aware of the auditory stimuli during the night. The number of auditory stimuli that participants had guessed were replayed in sleep (mean ± SEM, cued sounds = 3.82 ± 0.62; noncued sounds = 3.77 ± 0.63; cued spoken words = 2.73 ± 0.72; noncued spoken words = 2.68 ± 0.58) were applied to a 2 (TMR: On/Off) × 2 (Type: Verbal/Nonverbal) repeated measures ANOVA (again restricted to paired associates that participants had correctly recalled in the presleep test). There was no main effect of TMR (*F*(1, 22) = 0.01; *p =* .92) and no interaction between factors (*F*(1, 22) = 0.00; *p =* 1.00), indicating that participants were unable to categorize cued and noncued stimuli correctly. A main effect of Type (*F*(1, 22) = 7.93; *p =* .01) implied that participants had an overall greater tendency to guess that sounds were replayed in sleep than spoken words, irrespective of whether TMR had or had not taken place.

## DISCUSSION

We carried out two TMR experiments to investigate the cognitive mechanisms by which memories are retrieved for reactivation in SWS. In Experiment 1, verbal and nonverbal TMR with cues that were acoustically identical to training reduced forgetting of respective cued (vs. noncued) speech-word pairs and sound-word pairs to similar extents. In Experiment 2, nonverbal TMR with identical cues also reduced forgetting of cued (vs. noncued) sound-word pairs, replicating Experiment 1. However, verbal TMR with nonidentical cues (ie, presented in a different voice) appeared to reduce forgetting of both cued and noncued speech-word pairs. We observed no benefit of TMR for the spatial locations of the paired associates in either Experiment 1 or 2.

### Verbal Versus Nonverbal TMR

The findings of Experiment 1 are in keeping with a growing literature, which indicates that TMR delivered in SWS selectively enhances the retention of cued (vs. noncued) memories. Whereas previous studies have investigated separately the memory effects of TMR delivered with nonverbal cues^16–19,21^ and complex verbal cues,^26,28–31^ we carried out the first direct comparison of verbal and nonverbal TMR. Interestingly, verbal TMR reduced forgetting of cued (vs. noncued) paired associates to almost the same extent as nonverbal TMR, demonstrating that the memory benefits of these techniques are equivalent when the reactivation cues are identical to training.

Because spoken words are more abstract and complex stimuli than environmental sounds, with their wakeful perception requiring multiple stages of phonological and semantic analysis, one might have expected the memory benefits of verbal TMR to be of a smaller magnitude to those arising from nonverbal TMR. However, the findings of Experiment 1 suggest that the sleeping brain can exploit verbal and nonverbal materials for memory retrieval to comparable extents. One possible interpretation of this outcome is that the associations formed before sleep, regardless of whether they involve spoken words or environmental sounds, can be retrieved during sleep via a surface-level matching process that represents the form of the cues in a relatively concrete, unanalyzed acoustic manner. This would fit with a substantial body of evidence suggesting that recently encountered words are stored in an episodic form that tends to preserve nonlinguistic detail.^36,37^

The memory-enhancing effects of nonverbal TMR have been observed across a number of memory domains, including declarative memory,^16–20^ procedural memory,^21–24^ and emotional memory.^25,26^ The effects of verbal TMR have been demonstrated in language learning^28–30^ and, more recently, interpretation bias.^31,52^ Our observation that verbal TMR reduces forgetting of unrelated speech-word pairs provides new scope on the possible applications of this technique in experimental memory research. This may have wider implications for studies of memory encoding in sleep, which have thus far employed only nonverbal tones.^53^

### Two Processing Routes for Memory Retrieval in SWS

Given our interpretation of Experiment 1 as suggesting that cueing in sleep enables covert memory retrieval through a rather simple and superficial matching to recent episodic memories, one might expect that a switch to a different speaker in Experiment 2 would lead to a loss of the TMR benefit for verbal cues. In some ways, this prediction was upheld: whereas sound cues showed a near identical nonverbal TMR effect to Experiment 1, the verbal TMR effect for spoken word cues was notably absent. However, closer inspection of this finding revealed that the reason for the lack of a verbal TMR effect was not a heightened forgetting in the cued condition. Instead, it was a lower level of forgetting in the noncued condition. Our data therefore suggest that verbal TMR with nonidentical cues in Experiment 2 effectively enhanced the retention of both cued and noncued speech-word pairs. In other words, the spoken words for half of the speech-word pairs in Experiment 2 appeared to function as cues to the whole category of speech-associated items. TMR with nonidentical verbal cues may therefore elicit a generalized retention benefit across all categorically relevant memories.

Generalized, categorical memory benefits of TMR have been observed in previous work. In a recent study, Oudiette et al.^17^ trained participants to form associations between sounds and object locations that were assigned to a low- or high-value category. When the sounds associated with half of the low-value object locations were replayed in SWS, location accuracy improved across all objects in the low-value category. The authors proposed that, owing to the formulation of categorically relevant memories, replaying sound cues associated with a subset of low-value items had triggered reactivation for the whole set of low-value representations. It is important to note, however, that the TMR cues used in Oudiette et al.^17^ were identical to the sounds presented at training.

Why then might verbal cues that have a close acoustic match to recently encoded episodic memories lead to a focused retention benefit within the cued items, whereas verbal cues that have a linguistic (phonemic) but not an acoustic match to such memories yield a more generalized benefit across the full set of items? We think that the contrast between the two sets of cueing effects may hint at the presence of more than one mechanism for memory retrieval in sleep and may link in with theories that maintain dual coding of words.^54,55^ In particular, there is substantial evidence that recently encountered words may be encoded in two forms, one being episodic and retaining speaker detail and the other being more abstract and speaker independent.^36,56^ Episodic memories are thought to be stored as composite representations that include multiple elements corresponding to various aspects of an experience,^57,58^ which, in the case of words, are predominantly based on speaker-specific representations.^36,59^ A partial memory cue can prompt retrieval of all additional elements of an episodic representation via pattern completion,^60^ a process that is thought to be a core function of the hippocampus.^61,62^ Verbal TMR with cues that are acoustically identical to training may therefore stimulate the offline retrieval of individual episodic and speaker-specific memories via pattern completion, selectively enhancing the consolidation of cued (vs. noncued) representations.

Verbal TMR with nonidentical cues, however, may be an insufficiently close match on an acoustic level to trigger the retrieval of the relevant individual episodic and speaker-specific memories via pattern completion. Given this failure of direct retrieval, secondary mechanisms may come into play, and these may depend on the more abstract representations of words. Verbal cues that do not have a strong acoustic overlap with any recently encoded memory should trigger a chain of speech perception and word recognition processes that occur automatically in wake but are also operational in sleep.^34,35^ Once the appropriate word has been identified at a more abstract level, the broader categorical association between this word and other words from the presleep exposure session may lead to their reactivation as a group rather than individually (including both cued and noncued memories). Thus, we suggest that memory cueing in sleep can operate in at least two ways. Where there is an acoustic match to a recently formed and specific episodic association then cueing will benefit just that individual memory,^16,18,19^ but where there is only a more abstract match to a lexical item from a coherent category of associations then that category may benefit as a whole, making the effects of cueing more similar to the generalized benefits of TMR with olfactory cues.^63–65^ It should be noted that, from this perspective, the control word “surface” will not elicit a broad cascade of memory reactivation in sleep as this word is not learned before sleep and is thus not categorically related to the other verbal materials. While highly speculative, this interpretation of our data is aligned with spreading activation accounts of memory retrieval,^66^ which can explain the “fan effect” of increasing associated links within a memory network.^67^ It is important to note that by this view such extensive activation is only likely to be influential when there is no direct link between the verbal cue and the encoded representation mediating the spread of activation.

The proposals we have outlined are, as noted above, highly speculative and based on behavioral findings from only two experiments. Further investigation utilizing both behavioral and physiological research techniques is necessary to confirm these results and better understand how the properties of verbal stimuli influence the mechanisms of memory retrieval in sleep.

An alternative interpretation of our findings is that verbal TMR had no impact on the consolidation of speech-word pairs in Experiment 2, which may have been too strongly encoded to benefit from cueing in sleep. There are three key observations in our data that undermine this interpretation. First, presleep recall for the speech-word pairs and sound-word pairs was highly comparable in Experiment 2, meaning that differences in retention following verbal and nonverbal TMR cannot be attributed to unequal performance before sleep. Second, presleep recall for both the speech-word pairs and sound-word pairs was equivalent in Experiments 1 and 2, meaning that the diverse effects of verbal TMR with identical and nonidentical cues were unlikely to have arisen from group differences in memory accuracy. Third, while speech-word pairs were learned more quickly than sound-word pairs in Experiment 2, this was also the case in Experiment 1 and therefore cannot account for the differential impacts of verbal TMR observed in this study. Nevertheless, because we did not compare the effects of memory cueing in sleep with environmental sounds that were identical and nonidentical to training, we are unable to determine whether the observed effects of verbal TMR in Experiment 2 generalize to nonverbal TMR. Finally, it is notable that participants in Experiment 2 had a greater tendency to guess that sounds were replayed in sleep than spoken words. However, this effect was not modulated by the occurrence of TMR during sleep and is thus unlikely to be related to the differential impact of verbal and nonverbal TMR in Experiments 1 and 2.

### Spatial Memory

In contrast to previous work,^17–20,25^ we observed no benefit of TMR for spatial locations in either Experiment 1 or 2. There is a strong possibility that this incongruity arose from our instructions to participants regarding locations in the experimental task. During the training phase, participants were explicitly informed that memory for the word locations would not be included in their performance assessment. Consequently, while some participants performed very well in location recall before sleep, others performed very poorly. This variability may have prevented any spatial memory benefit of TMR from emerging in our data.

### Sleep Parameters

We observed no relationship between the memory benefits of TMR and spindle density in either Experiment 1 or 2. While this finding deviates from studies linking sleep spindles to successful memory cueing in sleep, these have typically employed procedural^21,22^ or spatial memory paradigms.^20,25^ One study has shown a relationship between TMR and paired associates retention, but this included only nonverbal auditory stimuli.^16^ Other studies have revealed transient changes in spindle activity following verbal cueing in sleep,^28,30^ but these occurred in the context of foreign language learning and not paired associates retention.

Although the correlation did not survive multiple comparisons correction, Experiment 1 revealed an intriguing relationship between time spent in REM and the memory benefits of nonverbal TMR (ie, for sound-word pairs). Consistent with this finding, a recent study by Tamminen et al.^68^ suggested that cueing newly learned memories in SWS supports the integration of those memories into existing knowledge during subsequent REM, supporting the view that SWS and REM hold complementary roles in overnight memory processing.^49^ However, Tamminen et al. addressed the lexical integration of novel words (eg, cathedruke), which were themselves replayed as TMR cues in sleep. One may therefore have expected time in REM in the current study to predict the TMR index for speech-word pairs, but this was not observed.

Again, while the correlation was below our multiple comparisons threshold, Experiment 2 revealed an inverse relationship between time spent in SWS and the memory effects of nonverbal TMR (with identical cues). This finding is in contrast to previous work suggesting that memories are selectively strengthened in SWS following TMR^19,25^ and opposes Active Systems accounts of the memory function of SWS.^7^ Notably, this relationship was not observed in Experiment 1, where both the verbal and nonverbal reactivation cues were identical to the stimuli presented at training (and the TMR index for sound-word pairs correlated instead with time in REM). The use of nonidentical verbal cues in Experiment 2 may have led to a different role of SWS in the consolidation of sound-word pairs following nonverbal TMR, though this is a speculative proposal to be addressed in future research.

### Future Considerations

Whether an auditory cue must be an integral component of a learned paired associate (eg, a speech/sound-word pair) in order for TMR to be effective is unknown. Previous studies that have used “reminder” cues (ie, auditory stimuli that are not an explicit part of the to-be-learned materials) have typically employed highly precise measures of memory accuracy (eg, object-location tasks that record performance changes in screen pixels^17–20^). It is therefore possible that the paired associates paradigm used in the current study is insensitive to reminder cues in sleep. However, recent work has shown that auditory reminder cues enhance recall of visually encoded picture-word associations,^31,52^ suggesting that the memory benefits of reminders may translate to verbal paired associates. Additional research is nevertheless required to determine how the manner in which a cue is linked to a newly learned memory influences the effectiveness of TMR. This is particularly important in terms of understanding the mechanisms by which identical and nonidentical cues influence consolidation in sleep.

In keeping with previous work,^16^ we opted to include only correctly recalled paired associates in the TMR and no TMR conditions and thereby addressed the effects of cueing on forgetting rates after sleep. A limitation of this approach, however, is that it prevented us from examining how TMR influences memories that are initially forgotten before sleep and whether cueing can “draw” such memories across a retrieval threshold. Indeed, recent work has suggested that TMR in sleep not only reduces forgetting but also increases the likelihood of “gaining” a memory that was previously forgotten.^69^ Addressing this question in the context of the current study would provide further important insights into the mechanisms of overnight memory processing.

### Summary and Conclusions

We carried out two experiments to investigate the cognitive mechanisms of memory retrieval in SWS. In Experiment 1, we compared the memory benefits of TMR with verbal and nonverbal cues that were identical to the stimuli encountered at training. We found that verbal and nonverbal TMR reduced forgetting of cued (vs. noncued) associations to similar extents, indicating that these techniques yield equal benefits for overnight consolidation. We re-examined verbal and nonverbal TMR in Experiment 2, but on this occasion, the gender of the verbal stimuli was switched between training and sleep (the nonverbal stimuli remained identical to training). Nonverbal TMR with identical cues reduced forgetting of cued (vs. noncued) associations, replicating Experiment 1. However, verbal TMR with nonidentical cues appeared to reduce forgetting of both cued and noncued speech-word pairs. These experiments provide the first evidence that the memory effects of verbal TMR are influenced by the acoustic overlap between spoken words delivered at training and in sleep. Although further investigation is required to confirm these results, our findings suggest that there may be two processing routes for memory retrieval in sleep. While TMR with acoustically identical cues may trigger the retrieval of individual memories via simple episodic matching, TMR with nonidentical cues may utilize linguistic decoding mechanisms, resulting in generalized retrieval across a broad category of interconnected memory traces.

## FUNDING

The research was funded by an Economic and Social Research Council grant (ES/I038586/1) awarded to MGG and SL.

## DISCLOSURE STATEMENT

None declared.

## References

[1] Gais S, Lucas B, Born J Sleep after learning aids memory recall. Learn Mem. 2006; 13(3): 259–262. 16741280 10.1101/lm.132106 PMC10807868

[2] Plihal W, Born J Effects of early and late nocturnal sleep on declarative and procedural memory. J Cogn Neurosci. 1997; 9(4): 534–547. 23968216 10.1162/jocn.1997.9.4.534

[3] Tucker MA, Hirota Y, Wamsley EJ, Lau H, Chaklader A, Fishbein W A daytime nap containing solely non-REM sleep enhances declarative but not procedural memory. Neurobiol Learn Mem. 2006; 86(2): 241–247. 16647282 10.1016/j.nlm.2006.03.005

[4] van der Helm E, Gujar N, Nishida M, Walker MP Sleep-dependent facilitation of episodic memory details. PLoS One. 2011; 6(11): e27421. 22114672 10.1371/journal.pone.0027421 PMC3219667

[5] Paller KA, Voss JL Memory reactivation and consolidation during sleep. Learn Mem. 2004; 11(6): 664–670. 15576883 10.1101/lm.75704 PMC534694

[6] Born J, Rasch B, Gais S Sleep to remember. Neuroscientist. 2006; 12(5): 410–424. 16957003 10.1177/1073858406292647

[7] Rasch B, Born J About sleep’s role in memory. Physiol Rev. 2013; 93(2): 681–766. 23589831 10.1152/physrev.00032.2012 PMC3768102

[8] Born J, Wilhelm I System consolidation of memory during sleep. Psychol Res. 2012; 76(2): 192–203. 21541757 10.1007/s00426-011-0335-6 PMC3278619

[9] Diekelmann S, Born J The memory function of sleep. Nat Rev Neurosci. 2010; 11(2): 114–126. 20046194 10.1038/nrn2762

[10] Lewis PA, Durrant SJ Overlapping memory replay during sleep builds cognitive schemata. Trends Cogn Sci. 2011; 15(8): 343–351. 21764357 10.1016/j.tics.2011.06.004

[11] Ji D, Wilson MA Coordinated memory replay in the visual cortex and hippocampus during sleep. Nat Neurosci. 2007; 10(1): 100–107. 17173043 10.1038/nn1825

[12] Peigneux P, Laureys S, Fuchs S Are spatial memories strengthened in the human hippocampus during slow wave sleep? Neuron. 2004; 44(3): 535–545. 15504332 10.1016/j.neuron.2004.10.007

[13] Wilson MA, McNaughton BL Reactivation of hippocampal ensemble memories during sleep. Science. 1994; 265(5172): 676–679. 8036517 10.1126/science.8036517

[14] Schouten DI, Pereira SIR, Tops M, Louzada FM State of the art on targeted memory reactivation: sleep your way to enhanced cognition. Sleep Med Rev. in press. 10.1016/j.smrv.2016.04.002 27296303

[15] Oudiette D, Paller KA Upgrading the sleeping brain with targeted memory reactivation. Trends Cogn Sci. 2013; 17(3): 142–149. 23433937 10.1016/j.tics.2013.01.006

[16] Fuentemilla L, Miró J, Ripollés P Hippocampus-dependent strengthening of targeted memories via reactivation during sleep in humans. Curr Biol. 2013; 23(18): 1769–1775. 24012316 10.1016/j.cub.2013.07.006

[17] Oudiette D, Antony JW, Creery JD, Paller KA The role of memory reactivation during wakefulness and sleep in determining which memories endure. J Neurosci. 2013; 33(15): 6672–6678. 23575863 10.1523/JNEUROSCI.5497-12.2013 PMC3677604

[18] Rudoy JD, Voss JL, Westerberg CE, Paller KA Strengthening individual memories by reactivating them during sleep. Science. 2009; 326(5956): 1079. 19965421 10.1126/science.1179013 PMC2990343

[19] Cairney SA, Lindsay S, Sobczak JM, Paller KA, Gaskell MG The benefits of targeted memory reactivation for consolidation in sleep are contingent on memory accuracy and direct cue-memory associations. Sleep. 2016; 39(5): 1139–1150. 26856905 10.5665/sleep.5772 PMC4835313

[20] Creery JD, Oudiette D, Antony JW, Paller KA Targeted memory reactivation during sleep depends on prior learning. Sleep. 2015; 38(5): 755–763. 25515103 10.5665/sleep.4670 PMC4402655

[21] Antony JW, Gobel EW, O’Hare JK, Reber PJ, Paller KA Cued memory reactivation during sleep influences skill learning. Nat Neurosci. 2012; 15(8): 1114–1116. 22751035 10.1038/nn.3152 PMC3498459

[22] Cousins JN, El-Deredy W, Parkes LM, Hennies N, Lewis PA Cued memory reactivation during slow-wave sleep promotes explicit knowledge of a motor sequence. J Neurosci. 2014; 34(48): 15870–15876. 25429129 10.1523/JNEUROSCI.1011-14.2014 PMC4244461

[23] Schönauer M, Geisler T, Gais S Strengthening procedural memories by reactivation in sleep. J Cogn Neurosci. 2014; 26(1): 143–153. 23984946 10.1162/jocn_a_00471

[24] Cousins JN, El-Deredy W, Parkes LM, Hennies N, Lewis PA Cued reactivation of motor learning during sleep leads to overnight changes in functional brain activity and connectivity. PLoS Biol. 2016; 14(5): e1002451. 27137944 10.1371/journal.pbio.1002451 PMC4854410

[25] Cairney SA, Durrant SJ, Hulleman J, Lewis PA Targeted memory reactivation during slow wave sleep facilitates emotional memory consolidation. Sleep. 2014; 37(4): 701–7, 707A. 24688163 10.5665/sleep.3572 PMC3954173

[26] Lehmann M, Schreiner T, Seifritz E, Rasch B Emotional arousal modulates oscillatory correlates of targeted memory reactivation during NREM, but not REM sleep. Sci Rep. 2016; 6: 39229. 27982120 10.1038/srep39229 PMC5159847

[27] Hu X, Antony JW, Creery JD, Vargas IM, Bodenhausen GV, Paller KA Cognitive neuroscience. Unlearning implicit social biases during sleep. Science. 2015; 348(6238): 1013–1015. 26023137 10.1126/science.aaa3841 PMC4467959

[28] Schreiner T, Rasch B Boosting vocabulary learning by verbal cueing during sleep. Cereb Cortex. 2015; 25(11): 4169–4179. 24962994 10.1093/cercor/bhu139

[29] Schreiner T, Göldi M, Rasch B Cueing vocabulary during sleep increases theta activity during later recognition testing. Psychophysiology. 2015; 52(11): 1538–1543. 26235609 10.1111/psyp.12505

[30] Schreiner T, Lehmann M, Rasch B Auditory feedback blocks memory benefits of cueing during sleep. Nat Commun. 2015; 6: 8729. 26507814 10.1038/ncomms9729 PMC4640077

[31] Groch S, McMakin D, Guggenbühl P, Rasch B, Huber R, Wilhelm I Memory cueing during sleep modifies the interpretation of ambiguous scenes in adolescents and adults. Dev Cogn Neurosci. 2016; 17: 10–18. 26588358 10.1016/j.dcn.2015.10.006 PMC6990077

[32] de Saussure F Nature of the linguistic sign. Cours de linguistique generale. In: Richter DH, ed. The Critical Tradition: Classic Texts and Contemporary Trends. 2nd ed: Boston: Bedford; 1916: 832–835.

[33] Gaskell MG, Mirković J, eds. Speech perception and spoken word recognition. Oxford: Psychology Press, 2016.

[34] Brualla J, Romero MF, Serrano M, Valdizán JR Auditory event-related potentials to semantic priming during sleep. Electroencephalogr Clin Neurophysiol. 1998; 108(3): 283–290. 9607517 10.1016/s0168-5597(97)00102-0

[35] Ibáñez A, López V, Cornejo C ERPs and contextual semantic discrimination: degrees of congruence in wakefulness and sleep. Brain Lang. 2006; 98(3): 264–275. 16782185 10.1016/j.bandl.2006.05.005

[36] Goldinger SD Words and voices: episodic traces in spoken word identification and recognition memory. J Exp Psychol Learn Mem Cogn. 1996; 22(5): 1166–1183. 8926483 10.1037//0278-7393.22.5.1166

[37] Goldinger SD Echoes of echoes? An episodic theory of lexical access. Psychol Rev. 1998; 105(2): 251–279. 9577239 10.1037/0033-295x.105.2.251

[38] Maki WS, McKinley LN, Thompson AG Semantic distance norms computed from an electronic dictionary (WordNet). Behav Res Methods Instrum Comput. 2004; 36(3): 421–431. 15641432 10.3758/bf03195590

[39] Nelson DL, McEvoy CL, Schreiber TA The University of South Florida word association, rhyme, and word fragment norms http://www.usf.edu/FreeAssociation/1998.

[40] Buysse DJ, Reynolds CF 3rd, Monk TH, Berman SR, Kupfer DJ The Pittsburgh Sleep Quality Index: a new instrument for psychiatric practice and research. Psychiatry Res. 1989; 28(2): 193–213. 2748771 10.1016/0165-1781(89)90047-4

[41] Hoddes E, Zarcone V, Smythe H, Phillips R, Dement WC Quantification of sleepiness: a new approach. Psychophysiology. 1973; 10(4): 431–436. 4719486 10.1111/j.1469-8986.1973.tb00801.x

[42] Wilhelm I, Diekelmann S, Molzow I, Ayoub A, Mölle M, Born J Sleep selectively enhances memory expected to be of future relevance. J Neurosci. 2011; 31(5): 1563–1569. 21289163 10.1523/JNEUROSCI.3575-10.2011 PMC6623736

[43] Iber C, Ancoli-Israel S, Chesson A, Quan SF. The AASM Manual for the Scoring of Sleep and Associated Events: Rules, Terminology and Technical Specification. Westchester (IL): American Academy of Sleep Medicine; 2007.

[44] Ferrarelli F, Huber R, Peterson MJ Reduced sleep spindle activity in schizophrenia patients. Am J Psychiatry. 2007; 164(3): 483–492. 17329474 10.1176/ajp.2007.164.3.483

[45] Cairney SA, Durrant SJ, Jackson R, Lewis PA Sleep spindles provide indirect support to the consolidation of emotional encoding contexts. Neuropsychologia. 2014; 63: 285–292. 25223465 10.1016/j.neuropsychologia.2014.09.016

[46] Tamminen J, Lambon Ralph MA, Lewis PA The role of sleep spindles and slow-wave activity in integrating new information in semantic memory. J Neurosci. 2013; 33(39): 15376–15381. 24068804 10.1523/JNEUROSCI.5093-12.2013 PMC3782619

[47] Tamminen J, Payne JD, Stickgold R, Wamsley EJ, Gaskell MG Sleep spindle activity is associated with the integration of new memories and existing knowledge. J Neurosci. 2010; 30(43): 14356–14360. 20980591 10.1523/JNEUROSCI.3028-10.2010 PMC2989532

[48] Weighall AR, Henderson LM, Barr DJ, Cairney SA, Gaskell MG Eye-tracking the time‐course of novel word learning and lexical competition in adults and children. Brain Lang. 2017; 167: 13–27. 27562102 10.1016/j.bandl.2016.07.010

[49] Cairney SA, Durrant SJ, Power R, Lewis PA Complementary roles of slow-wave sleep and rapid eye movement sleep in emotional memory consolidation. Cereb Cortex. 2015; 25(6): 1565–1575. 24408956 10.1093/cercor/bht349

[50] Durrant SJ, Cairney SA, Lewis PA Overnight consolidation aids the transfer of statistical knowledge from the medial temporal lobe to the striatum. Cereb Cortex. 2013; 23(10): 2467–2478. 22879350 10.1093/cercor/bhs244

[51] Durrant SJ, Taylor C, Cairney S, Lewis PA Sleep-dependent consolidation of statistical learning. Neuropsychologia. 2011; 49(5): 1322–1331. 21335017 10.1016/j.neuropsychologia.2011.02.015

[52] Groch S, Preiss A, McMakin DL Targeted reactivation during sleep differentially affects negative memories in socially anxious and healthy children and adolescents. J Neurosci. 2017; 37(9): 2425–2434. 28143960 10.1523/JNEUROSCI.1912-16.2017 PMC6596843

[53] Arzi A, Shedlesky L, Ben-Shaul M Humans can learn new information during sleep. Nat Neurosci. 2012; 15(10): 1460–1465. 22922782 10.1038/nn.3193

[54] Davis MH, Gaskell MG A complementary systems account of word learning: neural and behavioural evidence. Philos Trans R Soc Lond B Biol Sci. 2009; 364(1536): 3773–3800. 19933145 10.1098/rstb.2009.0111 PMC2846311

[55] Gow DWJr. The cortical organization of lexical knowledge: a dual lexicon model of spoken language processing. Brain Lang. 2012; 121(3): 273–288. 22498237 10.1016/j.bandl.2012.03.005 PMC3348354

[56] McQueen JM, Cutler A, Norris D Phonological abstraction in the mental lexicon. Cogn Sci. 2006; 30(6): 1113–1126. 21702849 10.1207/s15516709cog0000_79

[57] Gardner-Medwin AR The recall of events through the learning of associations between their parts. Proc R Soc Lond B Biol Sci. 1976; 194(1116): 375–402. 11493 10.1098/rspb.1976.0084

[58] Marr D Simple memory: a theory for archicortex. Philos Trans R Soc Lond B Biol Sci. 1971; 262(841): 23–81. 4399412 10.1098/rstb.1971.0078

[59] McLennan CT, Luce PA Examining the time course of indexical specificity effects in spoken word recognition. J Exp Psychol Learn Mem Cogn. 2005; 31(2): 306–321. 15755247 10.1037/0278-7393.31.2.306

[60] Horner AJ, Burgess N Pattern completion in multielement event engrams. Curr Biol. 2014; 24(9): 988–992. 24746796 10.1016/j.cub.2014.03.012 PMC4012134

[61] Nakazawa K, Quirk MC, Chitwood RA Requirement for hippocampal CA3 NMDA receptors in associative memory recall. Science. 2002; 297(5579): 211–218. 12040087 10.1126/science.1071795 PMC2877140

[62] Wills TJ, Lever C, Cacucci F, Burgess N, O’Keefe J Attractor dynamics in the hippocampal representation of the local environment. Science. 2005; 308(5723): 873–876. 15879220 10.1126/science.1108905 PMC2680068

[63] Diekelmann S, Büchel C, Born J, Rasch B Labile or stable: opposing consequences for memory when reactivated during waking and sleep. Nat Neurosci. 2011; 14(3): 381–386. 21258327 10.1038/nn.2744

[64] Rasch B, Büchel C, Gais S, Born J Odor cues during slow-wave sleep prompt declarative memory consolidation. Science. 2007; 315(5817): 1426–1429. 17347444 10.1126/science.1138581

[65] Rihm JS, Diekelmann S, Born J, Rasch B Reactivating memories during sleep by odors: odor specificity and associated changes in sleep oscillations. J Cogn Neurosci. 2014; 26(8): 1806–1818. 24456392 10.1162/jocn_a_00579

[66] Anderson JR A spreading activation theory of memory. J Verbal Learn Verbal Behav. 1983; 22: 261–295.

[67] Anderson JR Retrieval of propositional information from long-term memory. Cogn Psychol. 1974; 6: 451–474.

[68] Tamminen J, Lambon Ralph MA, Lewis PA Targeted memory reactivation of newly learned words during sleep triggers REM-mediated integration of new memories and existing knowledge. Neurobiol Learn Mem. 2017; 137: 77–82. 27864086 10.1016/j.nlm.2016.11.012

[69] Schreiner T, Rasch B To gain or not to gain – the complex role of sleep for memory: comment on Dumay (2016). Cortex. 2016. doi: 10.1016/j.cortex.2016.06.011. 10.1016/j.cortex.2016.06.011 27423210

